# (*E*)-3-(2,6-Dichloro­benzyl­idene)indolin-2-one

**DOI:** 10.1107/S1600536809043487

**Published:** 2009-10-28

**Authors:** Hongming Zhang, Haribabu Ankati, Ed Biehl

**Affiliations:** a Department of Chemistry, Southern Methodist University, Dallas, TX 75275, USA

## Abstract

There are two independent mol­ecules in the asymmetric unit of the title compound, C_15_H_9_Cl_2_NO. The dihedral angles between the oxindolyl and dichloro­phenyl rings are essentially identical for the two independent mol­ecules [63.4 (1) and 63.2 (1)°]. Dimers linked by amide–carbonyl N—H⋯O hydrogen bonds are formed from each symmetry-independent mol­ecule and the respective symmetry equivalent created by inversion.

## Related literature

For the syntheses and structures of related compounds, see: Ankati *et al.* (2009 ▶); Zhang *et al.* (2008 ▶, 2009*a* ▶,*b* ▶,*c* ▶). For the pharmacological properties of 3-(substituted-benzyl­idene)-1,3-dihydro-indolin derivatives, see: Andreani *et al.* (2006 ▶); Balderamos *et al.* (2008 ▶); Johnson *et al.* (2005 ▶); Olgen *et al.* (2005 ▶, 2007 ▶); Sun *et al.* (2003 ▶)
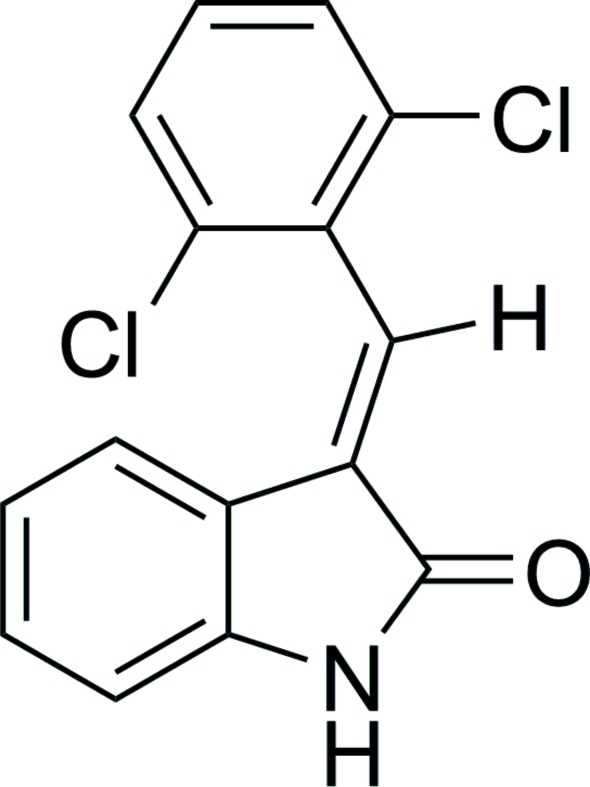


## Experimental

### 

#### Crystal data

C_15_H_9_Cl_2_NO
*M* *_r_* = 290.13
Triclinic, 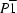

*a* = 8.3908 (5) Å
*b* = 12.6079 (7) Å
*c* = 12.7635 (7) Å
α = 99.334 (1)°
β = 91.188 (1)°
γ = 96.338 (1)°
*V* = 1323.2 (1) Å^3^
*Z* = 4
Mo *K*α radiation
μ = 0.48 mm^−1^
*T* = 296 K
0.35 × 0.17 × 0.08 mm

#### Data collection

Bruker APEX diffractometer
Absorption correction: multi-scan (*SADABS*; Sheldrick, 1996 ▶) *T*_min_ = 0.849, *T*_max_ = 0.964
16946 measured reflections
6459 independent reflections
4669 reflections with *I* > 2σ(*I*)
*R*_int_ = 0.024

#### Refinement

*R*[*F* ^2^ > 2σ(*F* ^2^)] = 0.052
*wR*(*F* ^2^) = 0.134
*S* = 1.03
6459 reflections
343 parameters
H-atom parameters constrained
Δρ_max_ = 0.33 e Å^−3^
Δρ_min_ = −0.23 e Å^−3^

### 

Data collection: *SMART* (Bruker, 1997 ▶); cell refinement: *SAINT* (Bruker, 1997 ▶); data reduction: *SAINT*; program(s) used to solve structure: *SHELXS97* (Sheldrick, 2008 ▶); program(s) used to refine structure: *SHELXL97* (Sheldrick, 2008 ▶); molecular graphics: *SHELXTL* (Sheldrick, 2008 ▶); software used to prepare material for publication: *SHELXTL* and *publCIF* (Westrip, 2009 ▶).

## Supplementary Material

Crystal structure: contains datablocks I, global. DOI: 10.1107/S1600536809043487/im2152sup1.cif

Structure factors: contains datablocks I. DOI: 10.1107/S1600536809043487/im2152Isup2.hkl

Additional supplementary materials:  crystallographic information; 3D view; checkCIF report

## Figures and Tables

**Table 1:** Hydrogen-bond geometry (Å, °)

*D*—H⋯*A*	*D*—H	H⋯*A*	*D*⋯*A*	*D*—H⋯*A*
N21—H21⋯O22^i^	0.86	2.03	2.854 (2)	159
N1—H1⋯O2^ii^	0.86	1.99	2.837 (2)	171

Symmetry codes: (i) 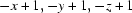; (ii) 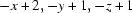.

**Fig. 1.**
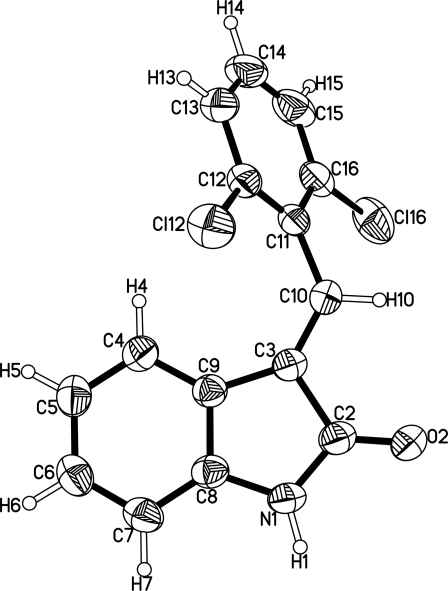
: A view of one of the independent molecules with displacement ellipsoids drawn at the 40% probability level. H atoms are presented as open circles with arbitrary radii. Atoms of another independent molecule were labeled as N21 H21 C22 O22 through C36 Cl36.

**Fig. 2.**
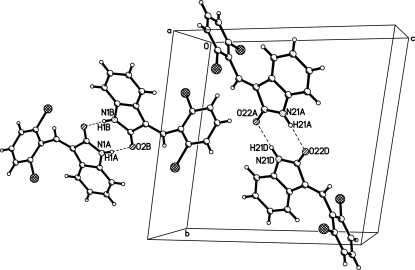
: A unit cell packing view of the title compound. Dashed lines indicate hydrogen bonds. For clarity, H atoms are presented as open circles with arbitrary radii.

## supplementary crystallographic information

### Comment

3-(Substituted-benzylidene)-1,3-dihydro-indolin derivatives show a variety of pharmacologically important properties such as being protein and tyrosine kinase inhibitors (Olgen *et al.*, 2005, 2007; Sun *et al.*, 2003) as well as antitumor (Andreani *et al.*, 2006) and neuroprotective agents (Johnson *et al.*, 2005). We have designed, synthesized and crystallized several 3-substituted indolin-2-one derivatives to study their neuroprotective properties (Balderamos *et al.*, 2008 and Ankati *et al.*, 2009). In relation of structure-activity of 3-substituted indolin-2-ones, the title compound was synthesized and its crystal structure is reported here. It is similar to the structure of 5-bromo substituted (*E*)-5-bromo-3-(2,6-dichlorobenzylidene)indolin-2-one (Zhang *et al.* 2009*c*). The X-ray crystal structure shows the title compound to show an *E* configuration.

For studying the biological properties a series of 3-substituted indolin-2-one derivatives have been synthesized in our lab and their neuroprotective activities have been tested (Balderamos *et al.* 2008). As a part of our research on the relationship between the biological activities and solid structures a couple of crystal structures of the derivatives have been carried out (Zhang, *et al.*, 2008, 2009*a*, 2009*b*, 2009*c*). The title compound consists of an oxindolyl and a dichlorophenyl unit (Fig 1). The dihedral angles between the two aromatic rings are basically identical for the two independent molecules and measure to 63.4°(1) and 63.2°(1), respectively. The crystal structure revealed that intermolecular H-bonds (Table 1), linking two symmetry related inverted molecules, form an eight membered dimeric ring system (Fig 2).

### Experimental

The title compound was synthesized by the condensation of 2,6-dichlorobenzaldehyde (1 mmol) with 2-oxindole (1 mmol) in ethanol (10 ml) in the presence of catalytic amount of piperidine (0.1 mmol) with a yield of 83%. After refluxing for 3 hr, the reaction mixture was left to stand overnight. The resulting crude solid was filtered, washed with cold ethanol (10 ml) and dried. Red single crystals of the compound suitable for X-ray structure determination obtained by recrystallization from ethanol.

### Refinement

All H atom were placed in calculated positions and included in the final cycles of refinement using a riding model, with distances N–H = 0.86 Å and C–H = 0.93 Å, and displacement parameters *U*_ĩso_(H) = 1.2*U*_eq_(N,*C*).

### Crystal data

**Table:**

C_15_H_9_Cl_2_NO	*Z* = 4
*M**_r_* = 290.13	*F*(000) = 592
Triclinic, *P*1	*D*_x_ = 1.456 Mg m^−^^3^
Hall symbol: -P 1	Mo *K*α radiation, λ = 0.71073 Å
*a* = 8.3908 (5) Å	Cell parameters from 6257 reflections
*b* = 12.6079 (7) Å	θ = 2.8–27.9°
*c* = 12.7635 (7) Å	µ = 0.48 mm^−^^1^
α = 99.334 (1)°	*T* = 296 K
β = 91.188 (1)°	Plates, orange
γ = 96.338 (1)°	0.35 × 0.17 × 0.08 mm
*V* = 1323.2 (1) Å^3^	

### Data collection

**Table:**

Bruker APEX diffractometer	6459 independent reflections
Radiation source: fine-focus sealed tube	4669 reflections with *I* > 2σ(*I*)
graphite	*R*_int_ = 0.024
Detector resolution: 83.33 pixels mm^-1^	θ_max_ = 28.3°, θ_min_ = 1.6°
φ and ω scans	*h* = −11→11
Absorption correction: multi-scan (*SADABS*; Sheldrick, 1996)	*k* = −16→16
*T*_min_ = 0.849, *T*_max_ = 0.964	*l* = −16→16
16946 measured reflections	

### Refinement

**Table:**

Refinement on *F*^2^	Primary atom site location: structure-invariant direct methods
Least-squares matrix: full	Secondary atom site location: difference Fourier map
*R*[*F*^2^ > 2σ(*F*^2^)] = 0.052	Hydrogen site location: inferred from neighbouring sites
*wR*(*F*^2^) = 0.134	H-atom parameters constrained
*S* = 1.03	*w* = 1/[σ^2^(*F*_o_^2^) + (0.0619*P*)^2^ + 0.2428*P*] where *P* = (*F*_o_^2^ + 2*F*_c_^2^)/3
6459 reflections	(Δ/σ)_max_ < 0.001
343 parameters	Δρ_max_ = 0.33 e Å^−^^3^
0 restraints	Δρ_min_ = −0.23 e Å^−^^3^

### Special details

**Table:**

Geometry. All e.s.d.'s (except the e.s.d. in the dihedral angle between two l.s. planes) are estimated using the full covariance matrix. The cell e.s.d.'s are taken into account individually in the estimation of e.s.d.'s in distances, angles and torsion angles; correlations between e.s.d.'s in cell parameters are only used when they are defined by crystal symmetry. An approximate (isotropic) treatment of cell e.s.d.'s is used for estimating e.s.d.'s involving l.s. planes.
Refinement. Refinement of *F*^2^ against ALL reflections. The weighted *R*-factor *wR* and goodness of fit *S* are based on *F*^2^, conventional *R*-factors *R* are based on *F*, with *F* set to zero for negative *F*^2^. The threshold expression of *F*^2^ > σ(*F*^2^) is used only for calculating *R*-factors(gt) *etc*. and is not relevant to the choice of reflections for refinement. *R*-factors based on *F*^2^ are statistically about twice as large as those based on *F*, and *R*- factors based on ALL data will be even larger.

### Fractional atomic coordinates and isotropic or equivalent isotropic displacement parameters (Å^2^)

**Table:**

	*x*	*y*	*z*	*U*_iso_*/*U*_eq_	
N1	0.8867 (2)	0.59180 (14)	0.44745 (13)	0.0561 (4)	
H1	0.9144	0.5842	0.5108	0.067*	
C2	0.9182 (3)	0.52439 (18)	0.35853 (16)	0.0548 (5)	
O2	0.9914 (2)	0.44512 (13)	0.35421 (12)	0.0729 (5)	
C3	0.8476 (2)	0.56692 (16)	0.26602 (15)	0.0480 (5)	
C4	0.6896 (3)	0.73604 (17)	0.27271 (17)	0.0537 (5)	
H4	0.6677	0.7285	0.2000	0.064*	
C5	0.6374 (3)	0.82040 (18)	0.3414 (2)	0.0609 (6)	
H5	0.5800	0.8698	0.3146	0.073*	
C6	0.6698 (3)	0.83215 (18)	0.44947 (19)	0.0613 (6)	
H6	0.6343	0.8898	0.4944	0.074*	
C7	0.7538 (3)	0.76009 (18)	0.49217 (17)	0.0575 (5)	
H7	0.7760	0.7684	0.5649	0.069*	
C8	0.8033 (2)	0.67554 (16)	0.42338 (16)	0.0487 (5)	
C9	0.7750 (2)	0.66252 (16)	0.31353 (15)	0.0461 (4)	
C10	0.8627 (3)	0.51431 (17)	0.16770 (16)	0.0520 (5)	
H10	0.9152	0.4526	0.1636	0.062*	
C11	0.8090 (2)	0.53923 (16)	0.06520 (15)	0.0478 (5)	
C12	0.8584 (2)	0.63401 (17)	0.02580 (16)	0.0511 (5)	
Cl12	0.99044 (8)	0.73448 (5)	0.10073 (5)	0.07059 (19)	
C13	0.8105 (3)	0.6502 (2)	−0.07446 (18)	0.0665 (6)	
H13	0.8441	0.7148	−0.0979	0.080*	
C14	0.7137 (3)	0.5705 (3)	−0.13847 (19)	0.0764 (8)	
H14	0.6818	0.5810	−0.2059	0.092*	
C15	0.6628 (3)	0.4751 (2)	−0.10437 (19)	0.0747 (7)	
H15	0.5965	0.4211	−0.1482	0.090*	
C16	0.7111 (3)	0.46028 (18)	−0.00474 (17)	0.0570 (5)	
Cl16	0.64687 (9)	0.33919 (5)	0.03774 (6)	0.0858 (2)	
N21	0.6215 (2)	0.38621 (13)	0.51019 (13)	0.0534 (4)	
H21	0.6146	0.4447	0.5543	0.064*	
C22	0.5593 (3)	0.36652 (16)	0.40938 (16)	0.0513 (5)	
O22	0.4820 (2)	0.42554 (12)	0.36706 (12)	0.0687 (5)	
C23	0.6022 (2)	0.25727 (15)	0.36049 (15)	0.0461 (4)	
C24	0.7577 (3)	0.12452 (17)	0.44962 (18)	0.0558 (5)	
H24	0.7547	0.0704	0.3904	0.067*	
C25	0.8278 (3)	0.1109 (2)	0.5444 (2)	0.0666 (6)	
H25	0.8720	0.0473	0.5491	0.080*	
C26	0.8328 (3)	0.1911 (2)	0.6324 (2)	0.0684 (6)	
H26	0.8808	0.1805	0.6957	0.082*	
C27	0.7682 (3)	0.28678 (19)	0.62897 (17)	0.0602 (6)	
H27	0.7717	0.3405	0.6885	0.072*	
C28	0.6985 (2)	0.29923 (16)	0.53382 (16)	0.0491 (5)	
C29	0.6920 (2)	0.21915 (15)	0.44319 (15)	0.0461 (4)	
C30	0.5491 (2)	0.21366 (15)	0.26238 (15)	0.0481 (5)	
H30	0.4870	0.2549	0.2276	0.058*	
C31	0.5768 (2)	0.10769 (15)	0.20232 (14)	0.0461 (4)	
C32	0.4491 (3)	0.02878 (16)	0.16894 (16)	0.0518 (5)	
Cl32	0.25656 (7)	0.05641 (5)	0.20230 (6)	0.0764 (2)	
C33	0.4683 (3)	−0.07053 (18)	0.10949 (18)	0.0655 (6)	
H33	0.3801	−0.1218	0.0890	0.079*	
C34	0.6194 (4)	−0.0920 (2)	0.08132 (18)	0.0710 (7)	
H34	0.6337	−0.1587	0.0415	0.085*	
C35	0.7498 (3)	−0.0170 (2)	0.11093 (19)	0.0692 (7)	
H35	0.8519	−0.0322	0.0910	0.083*	
C36	0.7281 (3)	0.08203 (18)	0.17090 (17)	0.0562 (5)	
Cl36	0.89345 (8)	0.17731 (6)	0.20621 (6)	0.0860 (2)	

### Atomic displacement parameters (Å^2^)

**Table:**

	*U*^11^	*U*^22^	*U*^33^	*U*^12^	*U*^13^	*U*^23^
N1	0.0754 (12)	0.0573 (11)	0.0375 (9)	0.0106 (9)	−0.0024 (8)	0.0117 (8)
C2	0.0685 (14)	0.0529 (12)	0.0444 (11)	0.0080 (10)	−0.0019 (10)	0.0122 (9)
O2	0.1095 (14)	0.0638 (10)	0.0513 (9)	0.0352 (10)	−0.0080 (9)	0.0110 (7)
C3	0.0548 (11)	0.0485 (11)	0.0418 (10)	0.0073 (9)	−0.0001 (8)	0.0099 (8)
C4	0.0568 (12)	0.0541 (12)	0.0520 (12)	0.0080 (10)	0.0024 (9)	0.0128 (10)
C5	0.0615 (13)	0.0540 (13)	0.0704 (15)	0.0139 (10)	0.0091 (11)	0.0136 (11)
C6	0.0663 (14)	0.0517 (12)	0.0647 (15)	0.0066 (11)	0.0176 (11)	0.0042 (11)
C7	0.0677 (14)	0.0572 (13)	0.0451 (11)	0.0011 (11)	0.0086 (10)	0.0043 (10)
C8	0.0525 (11)	0.0490 (11)	0.0443 (11)	−0.0009 (9)	0.0015 (8)	0.0116 (9)
C9	0.0490 (11)	0.0462 (11)	0.0434 (10)	0.0035 (8)	0.0030 (8)	0.0095 (8)
C10	0.0614 (13)	0.0488 (11)	0.0481 (11)	0.0164 (10)	−0.0007 (9)	0.0084 (9)
C11	0.0518 (11)	0.0534 (12)	0.0386 (10)	0.0184 (9)	0.0017 (8)	0.0004 (8)
C12	0.0529 (12)	0.0584 (12)	0.0437 (11)	0.0180 (10)	0.0048 (9)	0.0053 (9)
Cl12	0.0724 (4)	0.0672 (4)	0.0680 (4)	−0.0048 (3)	0.0039 (3)	0.0073 (3)
C13	0.0767 (16)	0.0827 (17)	0.0482 (13)	0.0330 (14)	0.0112 (11)	0.0175 (12)
C14	0.0839 (18)	0.109 (2)	0.0389 (12)	0.0392 (17)	−0.0063 (11)	0.0035 (13)
C15	0.0667 (15)	0.097 (2)	0.0513 (14)	0.0177 (14)	−0.0086 (11)	−0.0180 (14)
C16	0.0572 (12)	0.0594 (13)	0.0507 (12)	0.0134 (10)	0.0053 (10)	−0.0062 (10)
Cl16	0.0954 (5)	0.0576 (4)	0.0953 (5)	−0.0016 (3)	0.0205 (4)	−0.0090 (3)
N21	0.0712 (11)	0.0381 (9)	0.0460 (10)	0.0049 (8)	−0.0040 (8)	−0.0062 (7)
C22	0.0656 (13)	0.0391 (10)	0.0470 (11)	0.0052 (9)	0.0022 (9)	0.0009 (8)
O22	0.1060 (13)	0.0475 (8)	0.0536 (9)	0.0290 (9)	−0.0097 (8)	−0.0010 (7)
C23	0.0558 (11)	0.0354 (9)	0.0457 (11)	0.0053 (8)	0.0030 (9)	0.0020 (8)
C24	0.0636 (13)	0.0454 (11)	0.0568 (13)	0.0091 (10)	−0.0043 (10)	0.0029 (9)
C25	0.0698 (15)	0.0580 (14)	0.0739 (16)	0.0116 (11)	−0.0136 (12)	0.0158 (12)
C26	0.0715 (15)	0.0714 (16)	0.0608 (14)	0.0008 (12)	−0.0205 (12)	0.0145 (12)
C27	0.0659 (14)	0.0593 (13)	0.0485 (12)	−0.0037 (11)	−0.0092 (10)	−0.0024 (10)
C28	0.0502 (11)	0.0421 (10)	0.0509 (11)	−0.0026 (8)	−0.0017 (9)	0.0019 (8)
C29	0.0488 (11)	0.0423 (10)	0.0454 (10)	0.0008 (8)	−0.0010 (8)	0.0046 (8)
C30	0.0578 (12)	0.0420 (10)	0.0450 (11)	0.0141 (9)	0.0004 (9)	0.0038 (8)
C31	0.0609 (12)	0.0435 (10)	0.0346 (9)	0.0146 (9)	−0.0012 (8)	0.0030 (8)
C32	0.0658 (13)	0.0482 (11)	0.0414 (10)	0.0149 (10)	−0.0065 (9)	0.0028 (9)
Cl32	0.0616 (4)	0.0763 (4)	0.0860 (5)	0.0078 (3)	−0.0018 (3)	−0.0020 (3)
C33	0.0947 (18)	0.0446 (12)	0.0544 (13)	0.0137 (12)	−0.0129 (12)	−0.0020 (10)
C34	0.115 (2)	0.0513 (13)	0.0489 (13)	0.0359 (15)	−0.0008 (13)	−0.0040 (10)
C35	0.0865 (18)	0.0728 (16)	0.0558 (13)	0.0412 (14)	0.0129 (12)	0.0105 (12)
C36	0.0642 (13)	0.0562 (12)	0.0503 (12)	0.0174 (10)	0.0042 (10)	0.0082 (10)
Cl36	0.0609 (4)	0.0907 (5)	0.1046 (6)	0.0068 (3)	0.0163 (4)	0.0106 (4)

### Geometric parameters (Å, °)

**Table:**

N1—C2	1.353 (3)	N21—C22	1.353 (3)
N1—C8	1.399 (3)	N21—C28	1.403 (3)
N1—H1	0.8600	N21—H21	0.8600
C2—O2	1.224 (2)	C22—O22	1.220 (3)
C2—C3	1.510 (3)	C22—C23	1.502 (3)
C3—C10	1.336 (3)	C23—C30	1.330 (3)
C3—C9	1.460 (3)	C23—C29	1.457 (3)
C4—C5	1.381 (3)	C24—C25	1.378 (3)
C4—C9	1.390 (3)	C24—C29	1.382 (3)
C4—H4	0.9300	C24—H24	0.9300
C5—C6	1.381 (3)	C25—C26	1.381 (3)
C5—H5	0.9300	C25—H25	0.9300
C6—C7	1.380 (3)	C26—C27	1.383 (3)
C6—H6	0.9300	C26—H26	0.9300
C7—C8	1.375 (3)	C27—C28	1.376 (3)
C7—H7	0.9300	C27—H27	0.9300
C8—C9	1.397 (3)	C28—C29	1.402 (3)
C10—C11	1.468 (3)	C30—C31	1.473 (3)
C10—H10	0.9300	C30—H30	0.9300
C11—C12	1.395 (3)	C31—C32	1.389 (3)
C11—C16	1.401 (3)	C31—C36	1.395 (3)
C12—C13	1.386 (3)	C32—C33	1.382 (3)
C12—Cl12	1.732 (2)	C32—Cl32	1.737 (2)
C13—C14	1.365 (4)	C33—C34	1.369 (4)
C13—H13	0.9300	C33—H33	0.9300
C14—C15	1.372 (4)	C34—C35	1.369 (4)
C14—H14	0.9300	C34—H34	0.9300
C15—C16	1.374 (3)	C35—C36	1.387 (3)
C15—H15	0.9300	C35—H35	0.9300
C16—Cl16	1.736 (3)	C36—Cl36	1.733 (2)
			
C2—N1—C8	111.47 (17)	C22—N21—C28	111.48 (16)
C2—N1—H1	124.3	C22—N21—H21	124.3
C8—N1—H1	124.3	C28—N21—H21	124.3
O2—C2—N1	126.48 (19)	O22—C22—N21	126.61 (19)
O2—C2—C3	126.87 (19)	O22—C22—C23	126.87 (19)
N1—C2—C3	106.64 (18)	N21—C22—C23	106.52 (18)
C10—C3—C9	136.10 (19)	C30—C23—C29	133.96 (18)
C10—C3—C2	118.73 (18)	C30—C23—C22	120.05 (18)
C9—C3—C2	105.17 (17)	C29—C23—C22	105.84 (16)
C5—C4—C9	119.3 (2)	C25—C24—C29	119.5 (2)
C5—C4—H4	120.4	C25—C24—H24	120.2
C9—C4—H4	120.4	C29—C24—H24	120.2
C4—C5—C6	120.6 (2)	C24—C25—C26	120.4 (2)
C4—C5—H5	119.7	C24—C25—H25	119.8
C6—C5—H5	119.7	C26—C25—H25	119.8
C7—C6—C5	121.3 (2)	C25—C26—C27	121.7 (2)
C7—C6—H6	119.3	C25—C26—H26	119.2
C5—C6—H6	119.3	C27—C26—H26	119.2
C8—C7—C6	117.7 (2)	C28—C27—C26	117.3 (2)
C8—C7—H7	121.2	C28—C27—H27	121.4
C6—C7—H7	121.2	C26—C27—H27	121.4
C7—C8—C9	122.4 (2)	C27—C28—C29	122.23 (19)
C7—C8—N1	128.23 (19)	C27—C28—N21	128.52 (19)
C9—C8—N1	109.37 (17)	C29—C28—N21	109.23 (17)
C4—C9—C8	118.71 (19)	C24—C29—C28	118.90 (18)
C4—C9—C3	133.96 (19)	C24—C29—C23	134.09 (18)
C8—C9—C3	107.33 (17)	C28—C29—C23	106.90 (17)
C3—C10—C11	129.73 (19)	C23—C30—C31	127.71 (18)
C3—C10—H10	115.1	C23—C30—H30	116.1
C11—C10—H10	115.1	C31—C30—H30	116.1
C12—C11—C16	115.33 (19)	C32—C31—C36	116.07 (18)
C12—C11—C10	125.09 (19)	C32—C31—C30	120.65 (18)
C16—C11—C10	119.4 (2)	C36—C31—C30	123.21 (19)
C13—C12—C11	122.4 (2)	C33—C32—C31	122.9 (2)
C13—C12—Cl12	117.53 (19)	C33—C32—Cl32	118.52 (19)
C11—C12—Cl12	120.03 (16)	C31—C32—Cl32	118.61 (15)
C14—C13—C12	119.5 (2)	C34—C33—C32	118.8 (2)
C14—C13—H13	120.3	C34—C33—H33	120.6
C12—C13—H13	120.3	C32—C33—H33	120.6
C13—C14—C15	120.7 (2)	C33—C34—C35	121.0 (2)
C13—C14—H14	119.7	C33—C34—H34	119.5
C15—C14—H14	119.7	C35—C34—H34	119.5
C14—C15—C16	119.2 (2)	C34—C35—C36	119.3 (2)
C14—C15—H15	120.4	C34—C35—H35	120.3
C16—C15—H15	120.4	C36—C35—H35	120.3
C15—C16—C11	122.9 (2)	C35—C36—C31	122.0 (2)
C15—C16—Cl16	118.9 (2)	C35—C36—Cl36	118.96 (19)
C11—C16—Cl16	118.12 (17)	C31—C36—Cl36	119.08 (16)

### Hydrogen-bond geometry (Å, °)

**Table:**

*D*—H···*A*	*D*—H	H···*A*	*D*···*A*	*D*—H···*A*
N21—H21···O22^i^	0.86	2.03	2.854 (2)	159
N1—H1···O2^ii^	0.86	1.99	2.837 (2)	171

Symmetry codes: (i) −*x*+1, −*y*+1, −*z*+1; (ii) −*x*+2, −*y*+1, −*z*+1.
